# Applied Anatomy, Medical, Surgical, and Operative
*"Applied Anatomy, Medical, Surgical, and Operative." By J. McClachan, M.D. (Edinburgh : Messrs. E. and S. Livingstone).


**Published:** 1890-05-10

**Authors:** 


### THE PRACTITIONER'S BOOKSHELF.

[Books for Review should be sent to The Editor, The Lodge, Porchester Square, W.]

### APPLIED ANATOMY, MEDICAL, SURGICAL, AND OPERATIVE.*

The appearance of the third edition of this work obviously points to its having supplied an actual want, and to appreciation of its contents on the part of students of medicine.

The present edition is more than four times the size of the first, and its scope has been so much widened as to render the title somewhat misleading. Its contents would perhaps be better justified by calling the book a manual of regional surgery, for the great bulk of the book consists of pathology, symptoms, diagnosis, and treatment, illustrated by anatomical reference. It can be called applied anatomy only on the assumption that surgery is an application of anatomical knowledge.

The evolution of the book perhaps explains its weak points due to numerous successive additions. Certain chapters treat with very great detail the subjects to which they refer, while others are strikingly defective. As an illustration, the section on diseases of the hip might be compared with that dealing with the arteries, which extends over 120 pages. "By Morbus coxæ," we are told, "is meant strumous arthritis of the hip joint," and practically no other disease of the hip joint is dealt with. In describing dislocations of this joint again, although the rarer forms of dislocation are mentioned after the description of the commoner varieties, no mention is made, either here or under the heading of fractures of the pelvis, of dislocation in conjunction with fracture of the floor or rim of the acetabulum, accidents not of extreme rarity and of great importance from a diagnostic point of view. Again, no mention is made of the not very uncommon dislocation of the bones of the forearm backwards and forwards, after falls on the extended pronated hand, and little weight is placed on the comparative rarity of dislocation of the wrist. The mode of administration of chloroform is described in great detail and with much care; but, on the other hand, although the author states that ether is a desirable anæsthetic in some cases, and should always be ready, no details as to its administration are included, some objections to its general use only being furnished. Operations, especially amputations, are furnished in great profusion, and to counteract the difficulty thus presented to the student, *résumés* are occasionally given. These, however, scarcely cover the whole ground, and we take it that the student will have little predilection for an operation which he has been taught is "ineffective, tedious, and bloody," although experience has shown that the method of Antyllus (the operation referred to) is a useful one, and that moreover, in other regions than the axilla—notably when an aneurismal sac is fed by numerous branches, or in the cases of small traumatic aneurisms in any position. Criticisms of this nature might be multiplied, while, on the other hand, operations which have fallen into desuetude, such as Ogston's for knock knee, or Wood's radical cure of hernia, are treated of at some length. In the matter of the treatment of fractures, the views of the author on the desirability of simplicity in splints scarcely warrant him in his wholesale condemnation of such splints as Gordon's, Carr's, and Nelaton's; and certainly more weight should be given to the immediate treatment of fractures by the application of plaister of Paris splints, a method of extreme value, not only as a saving of time and expense in hospital practice, but from its almost universal applicability, invaluable in emergencies, rendering the country surgeon, with a tin box of plaister of Paris, a roll of house flannel, and a few bandages, independent of all other material or appliance.

Lastly, as to the applied anatomy, considering the title of this book it is certainly scarcely treated with the thoroughness which might be desired. Although much space is devoted to histology and embryology, many obvious points of coarse anatomy are absent; thus, neither in the description of fracture of the femur, nor under the section devoted to the femoral artery, is mention made of the danger of fractures of the bone in the neighbourhood of the middle and lower thirds, by injury of the artery or vein, leading to hæmorrhage or thrombosis of vein, and possible embolism. Again, in the enumeration of the muscles acting on the hip joint no mention is made of the secondary action of the adductors as flexors and external rotators, or of the extensor action of the adductor magnus. In other directions the anatomy is scarcely abreast of the time. Thus the old nomenclature is adhered to when the cranial nerves are spoken of. We are told that the cæcum is covered anteriorly and at the sides by the peritoneum; and, again, that the angle of the femur in old age drops nearly to a right angle.

The above criticisms point to the most marked defects in this book, in part due to a want of relative balance, and in part to the attempt to produce a work suitable for preparation for examinations rather than an aid to actual practice. On the other hand, it contains a large amount of useful information, and the descriptions of operations are not only well put and readily intelligible, but, as already remarked, abundant.

*"Applied Anatomy, Medical, Surgical, and Operative." By J. McClachan, M.D. (Edinburgh: Messrs. E. and S. Livingstone).

